# Out of Africa: Biogeography and diversification of the pantropical pond skater genus *Limnogonus* Stål, 1868 (Hemiptera: Gerridae)

**DOI:** 10.1002/ece3.2688

**Published:** 2017-01-01

**Authors:** Zhen Ye, Yahui Zhen, Yanyan Zhou, Wenjun Bu

**Affiliations:** ^1^ College of Environmental Science and EngineeringNankai UniversityTianjinChina; ^2^ College of Life SciencesInstitute of EntomologyNankai UniversityTianjinChina

**Keywords:** boreotropical migration, Gondwanan vicariance, *Limnogonus*, long‐distance dispersal, pantropical distribution

## Abstract

Gondwanan vicariance, long‐distance dispersal (LDD), and boreotropical migration have been proposed as alternative hypotheses explaining the pantropical distribution pattern of organisms. In this study, the historical biogeography of the pond skater genus *Limnogonus* was reconstructed to evaluate the impact of biogeographical scenarios in shaping their modern transoceanic disjunction. We sampled almost 65% of recognized *Limnogonus* species. Four DNA fragments including 69 sequences were used to reconstruct a phylogram. Divergence time was estimated using a Bayesian relaxed clock method and three fossil calibrations. Diversification dynamics and ancestral area reconstruction were investigated by using maximum likelihood and Bayesian approaches. Our results showed the crown group of *Limnogonus* originated and diversified in Africa in the early Eocene (49 Ma, HPD: 38–60 Ma), subsequently expanding into other regions *via* dispersal. The colonization of the New World originated from the Oriental Region probably *via* the Bering Land Bridge in the late Eocene. Two split events between the Old World and New World were identified: one between Neotropics and Oriental region around the middle Oligocene (30 Ma, HPD: 22–38 Ma), and the other between Neotropics and Africa during the middle Miocene (14 Ma, HPD: 8–21 Ma). The evolutionary history of *Limnogonus* involved two biogeographical processes. Gondwanan vicariance was not supported in our analyses. The diversification of *Limnogonus* among Africa, Oriental, and Neotropical regions corresponded with the age of land bridge connection and dispersed as a member associated with the broad boreotropical belt before local cooling (34 Ma). The current transoceanic disjunctions in *Limnogonus* could be better explained by the disruption of “mixed‐mesophytic” forest belt; however, the direct transoceanic LDD between the Neotropics and Africa could not be ruled out. In addition, the “LDD” model coupled with island hopping could be a reasonable explanation for the diversification of the Oriental and Australian regions during the Oligocene.

## 1 Introduction

Transoceanic disjunction at the species level within genus is one of the common distribution patterns in biogeographical studies, particularly among plate tectonics in tropical Africa, the Indo‐Australian archipelago, and South America (Rota, Peña, & Miller, 2016; Thorne, 1972; Toussaint et al., 2016). Three main biogeographical hypotheses have been involved in explaining this distribution pattern, which is especially observed in pantropical organism lineages operating at different time scales. One hypothesis is tectonic‐driven vicariance resulting from the breakup of the Gondwanan supercontinent (Givnish & Renner, 2004). Gondwanan vicariance has been demonstrated as playing a significant role in shaping the distribution of organisms in the Southern Hemisphere after Wegener's theory of plate tectonics was put forward in the second half of the 20th century (Wegener, 1912). In recent years, with the advent and widespread use of time‐calibrated molecular phylogenetics, a second hypothesis, long‐distance dispersal (LDD), has experienced a renaissance on account of inconsistent divergence times inferred from many cases with the proposed timing of Gondwanan breakup (Heaney, 2007; Wei et al., 2015). Furthermore, historical biogeographical studies of underlying dispersal routes for pantropical disjunctions have been mostly explained by direct transoceanic LDD with subsequent speciation, such as plants and flying animals. It has been documented in biogeographical studies of numerous plants that transoceanic track by winds or ocean currents were found in ferns (Wei et al., 2015), bryophytes (Scheben et al., 2016), and angiosperm taxa (Linder & Barker, 2014; Michalak, Zhang, & Renner, 2010). As flying birds and insects are capable of active dispersal abilities and can also be facilitated by wind or thermal uplifts to some extent (Chapman, Drake, & Reynolds, 2011; Rota et al., 2016), it is also easy to imagine that the direct transoceanic LDD in such groups has played a significant role in shaping their disjunct distribution. However, when considering extinction events in biogeographical studies (Liu & Schneider, 2013), especially when subtropical or tropical disjunctions *via* the “mixed‐mesophytic” forest belt migration were involved (Lavin & Luckow, 1993), a third hypothesis, the “boreotropical migration” model, should be considered, which states that organisms dispersed *via* the northern routes to colonize subtropical or tropical regions, followed by extinction in large regions of middle‐ to high‐latitude areas of the Northern Hemisphere. This hypothesis postulated that a continuous tropical forest belt throughout the Northern Hemisphere during the early Cenozoic allowed for intercontinental dispersals *via* the Bering Land Bridge (BLB) or North Atlantic Land Bridge (NALB). Global cooling after the early Oligocene (34 Ma) triggered local extinctions in middle‐ to high‐latitude areas or southward low‐latitude migrations of the forest belt, eventually leading to the disruption of the continuous forest belt and leaving isolated remnants of “mixed‐mesophytic” forest belts in relatively low‐latitude regions (Mao, Hao, Liu, Adams, & Milne, 2010; Morley, 2003). This appealing hypothesis explaining transoceanic disjunction had been demonstrated in some thermophilic plants and phytophagous insects, such as the pantropical fern genus *Diplazium* (Athyriaceae) (Wei et al., 2015), the Holarctic shrub genus *Juniperus* (Cupressaceae) (Mao et al., 2010), and the Holarctic aphid genus *Cinara* (Meseguer, d'acier, Genson, & Jousselin, 2015).

This study focused on the genus of pond skater *Limnogonus* (Hemiptera: Heteroptera: Gerridae), a common and well‐known semi‐aquatic insect group from lentic habitats comprised of 28 species and 4 subspecies throughout the world's subtropical and tropical areas (Andersen, 1995; Damgaard, Buzzetti, Mazzucconi, Weir, & Zettel, 2010; Damgaard, Moreira, Weir, & Zettel, 2014). Traditionally, *Limnogonus* had been divided into two subgenera, *Limnogonus* s.str. and *Limnogonoides* Poisson, 1965. The two subgenera were foremost distinguished on the basis of presence (*Limnogonus*) or absence (*Limnogonoides*) of two light elongated spots on the anterior part of the pronotum (Andersen, 1975; Damgaard et al., 2010). This genus has a pantropical distribution with 32.1% (nine species) residing in Africa, 17.9% (five species) in the Oriental Region, 28.6% (eight species) in the Australian Region, and 21.4% (six species) in Neotropics Region (Andersen, 1995; Damgaard et al., 2014). Two species (i.e., *L. fossarum* and *L. hungerfordi*) are distributed widely from the Oriental to Australian regions (Andersen, 1995). Recent studies have shed new light on the phylogeny of the genus *Limnogonus*. Damgaard et al. (2010, 2014) found that the two subgenera were not reciprocally monophyletic; instead, seven well‐supported clades have subsequently been recognized. The biogeographical implications of these results indicated that the genus *Limnogonus* was primarily an Old World group, and the colonization of the New World happened independently only in two clades (Damgaard et al., 2010). However, because of lacking molecular dating and biogeographical analyses, the timing and order of these events were not clear and biogeographical hypothesis that could reasonably explain the transoceanic disjunction of pantropical genus *Limnogonus* has not been vigorously tested prior to this study.

In this study, we used an almost 2/3 species‐level coverage of *Limnogonus* and various analytical methods to provide for the first time a robust fossil‐calibrated phylogenetic framework by combining three mitochondrial (COI, COII, and 16S) and one nuclear markers (28S). We further employed fossil‐calibrated divergence time estimates, diversification dynamic analyses, and ancestral area reconstruction, to explicitly test the alternative hypotheses mentioned above to the historical biogeography of *Limnogonus*.

## 2 Materials and methods

### 2.1 Taxa sampling and DNA sequences

Our ingroup sampling followed the classification of Andersen (1975, 1995), and the dataset included 18 species/subspecies, which represented 58% of the described subgenus *Limnogonus* s.str. species (11/19) and 67% of the described subgenus *Limnogonoides* species (6/9) (Table S1). Whenever possible we included multiple specimens of each species in order to represent a broad geographic coverage. We generated 21 new sequences representing two widespread species (*L. f. fossarum* and *L. nitidus*) that occurred in China and Vietnam (GenBank accession numbers: KY172123–KY172143). The newly derived data were combined with previously published molecular phylogenic data (Damgaard et al., 2010). We believed that our sampling adequately reflected global species diversities of the genus *Limnogonus*. The outgroups included seven species of other genera in Gerridae and two taxa from the closely related family Veliidae (Table S1), which were all from the previously published data (Damgaard, 2008b; Damgaard et al., 2014). Three mitochondrial (COI, COII, and 16S) and one nuclear (28S) DNA sequences were used for analyses. DNA extraction and PCR program were performed following the methods of Damgaard and Cognato (2003). Sequences were aligned with Muscle 3.7 (Edgar, 2004) multiple alignments under default settings and visually proofread in Bioedit 7.0 software (Hall, 1999). Aligned gene regions (COI, COII, 16S, and 28S) were concatenated in MEGA 6.06 (Tamura, Stecher, Peterson, Filipski, & Kumar, 2013). Voucher information and GenBank accession numbers are provided in Table S1.

### 2.2 Phylogenetic analyses

Phylogenetic analyses were performed using MrBayes 3.2.1 (Ronquist & Huelsenbeck, 2003) for Bayesian inference (BI) and raxmlGUI 1.5 (Silvestro & Michalak, 2012) for maximum likelihood (ML). The dataset was partitioned by genes, and MrModeltest 2.3 (Nylander, 2004) was used to determine the best‐fit model under the Akaike information criterion (AIC). The GTR+I+G model was selected both for each gene and for the combined dataset. In the BI analyses, the Markov chain Monte Carlo (MCMC) algorithm was run for 5,000,000 generations and sampled every 100 generations with four chains, starting from a random tree. Convergence of runs was assessed using Tracer 1.5 (Rambaut & Drummond, 2007). With the first 25% of trees discarded as burn‐in, Bayesian posterior probabilities were calculated for the majority consensus trees of the remaining trees. In the ML analyses, the GTR+I+G model was set for all partitions; 1,000 rapid bootstrap inferences were executed before a thorough ML search. Other parameters were kept at default.

### 2.3 Divergence time estimates

Divergence times were inferred with BEAST 2.3.2 (Bouckaert et al., 2014). We assigned a lognormal relaxed clock with uncorrelated rates to each clock model. The Tree Model was set to “Speciation: Birth‐Death Process.” The GTR+I+G substitution model with unlinked data partitions was chosen to account for the mitochondrial and nuclear data. Three fossil calibration points were used: (1) the age of the oldest known Gerroidea amber fossil *Cretogerris albianus* Perrichot (100.5–113 Ma) for the root, following Perrichot, Nel, and Neraudeau (2005) and Damgaard (2008a) with a normal prior distribution (mean: 106.75 Ma and a standard deviation of 6.7); (2) *Aquarius lunpolaensis* from the Miocene (5–25 Ma) in Tibet was convincingly assigned to the extant *Aquarius najas* species group (Andersen, 1998; Damgaard, 2008a). We accepted the assignment of the *A. lunpolaensis* fossil, and normal prior distribution (mean: 15 Ma, and a standard deviation of 5) was used to constrain the crown age of the *A. najas* species group; (3) The well‐preserved *Succineogerris larssoni* Andersen, 2000 from Eocene‐Oligocene (38–54 Ma) Baltic amber was morphologically similar to the extant genus *Neogerris* and was identified as a close relative of *Neogerris* (Andersen, 2000; Damgaard et al., 2014; Zettel & Heiss, 2011). Therefore, we used normal prior distribution (mean: 46 Ma, and a standard deviation of 4) to constrain the crown age of the genus *Neogerris*. The analyses were run for 100,000,000 generations and sampled for every 1,000 generations. Convergence was monitored using Tracer 1.5 (Rambaut & Drummond, 2007), and the effective sample sizes (ESSs) of all parameters exceeded the threshold of 200. TreeAnnotator (Bouckaert et al., 2014) was used to summarize the set of postburn‐in trees (with the first 25% samples discarded as burn‐in) and their parameters to produce a maximum clade credibility (MCC) chronogram, which showed the mean divergence time estimates with 95% high posterior density (HPD) intervals.

### 2.4 Diversification dynamics analyses

We constructed semi‐logarithmic lineage‐through‐time (LTT) plots by using the R package APE 3.3 (http://ape-package.ird.fr) to visualize temporal variations in diversification rates. A total of 1,000 trees were sampled randomly from the converged BEAST trees and used to calculate a 95% credibility interval. We applied the software BAMM 2.5.0 to estimate the dynamics of speciation and extinction rates through time and among lineages (Rabosky, 2014; Rabosky, Grundler, Anderson, & Larson, 2014). We conducted BAMM analysis with four reversible jump MCMC running for 10,000,000 generations and sampled for every 1,000 generations. The prior distributions on speciation (λ) and extinction (μ) rates were estimated in R package BAMM tools (Rabosky et al., 2014) (lambdaInitPrior = 5.77, lambdaShiftPrior = 0.011, muInitPrior = 5.76). Missing taxon sampling was considered with a sampling fraction set to 0.65 (17/26). The BAMM output files were also analyzed using the R package BAMMtools (Rabosky et al., 2014). The ESSs of all parameters were guaranteed for a minimum of 200. The dynamic rate variation among tree lineages was evaluated using the following approaches in BAMMtools: (1) The mean phylorate plot indicated distinct speciation rates by mapping colors to rates on all branches, (2) macro‐evolutionary cohort analysis displayed the pairwise probability that any two species shared a common macro‐evolutionary rate dynamic, and (3) the speciation rate, extinction rate, and net diversification rate of *Limnogonus* were extracted by rate‐through‐time analysis.

### 2.5 Ancestral area reconstruction

Ancestral areas were determined using a likelihood approach in the dispersal–extinction–cladogenesis (DEC) model implemented in RASP 3.1 (Yu, Harris, Blair, & He, 2015) with source code of the C++ Lagrange version (Smith, 2009). Analyses were based on the MCC tree from BEAST analyses. Species geographic distribution data were obtained from the literature (Andersen, 1975, 1995; Damgaard et al., 2014). The following four biogeographical areas were defined based on the paleogeographical history of the continents (Mao et al., 2012), with several alterations tailored to *Limnogonus* distributions: (A) Africa; (B) India, Indo‐China Peninsula, and Malesia; (C) New Guinea, Pacific islands, and Australia; and (D) Central America, Caribbean, and South America. We considered one “LDD” model with a high dispersal rate at any time slice and one “boreotropical migration” model at four time slices (0–5, 5–30, 30–45, and > 45 Ma). We scaled dispersal rates among the areas based on geological history, climate history, and the presence and dissolution of land bridges and island chains of the continents through time (Table S2) to reflect different opportunities for dispersal available to *Limnogonus* over time. Dispersal rates were scaled from 0.10 for well‐separated areas to 1.00 for contiguous landmasses (Table S2). The “LDD” model was compared using likelihood ratios, χ^2^, and AIC tests against the “boreotropical migration” model. As the widespread species of *Limnogonus* do not occur in more than two of the four regions that we defined, the maximum number of areas assignable to each node was set to two in all analyses.

## 3 Results

### 3.1 Phylogenetics and divergence times

The combined matrix included 69 sequences and 2,041 aligned sites (16S: 403 bp; COI+COII: 1,168 bp; 28S: 470 bp). The resolved topology (BI/ML) (Fig. S1) was largely consistent with a previous phylogenetic study (Damgaard et al., 2014). BI/ML reconstructions suggested that *Limnogonus* and *Neogerris* were each resolved as monophyletic entities (Fig. S1). Within *Limnogonus*, we found that the two subgenera, *Limnogonus* s.str. and *Limnogonoides*, were also not reciprocally monophyletic and recognized the same seven well‐supported clades (clades I–VII) from Damgaard et al. (2014) (Fig. S1). The only difference from Damgaard et al. (2014) was that the clade III comprising *L. franciscanus* from the New World and *L. cereiventris* from the Afrotropical Region were not placed as a sister group to the clade V, but placed as a sister group to clades I+II (Fig. S1). In addition, the newly collected data of *L. f. fossarum* populations from China (i.e., Hainan and Taiwan islands) were unexpectedly grouped with the remote Andaman Island population (Fig. S1).

**Figure 1:**
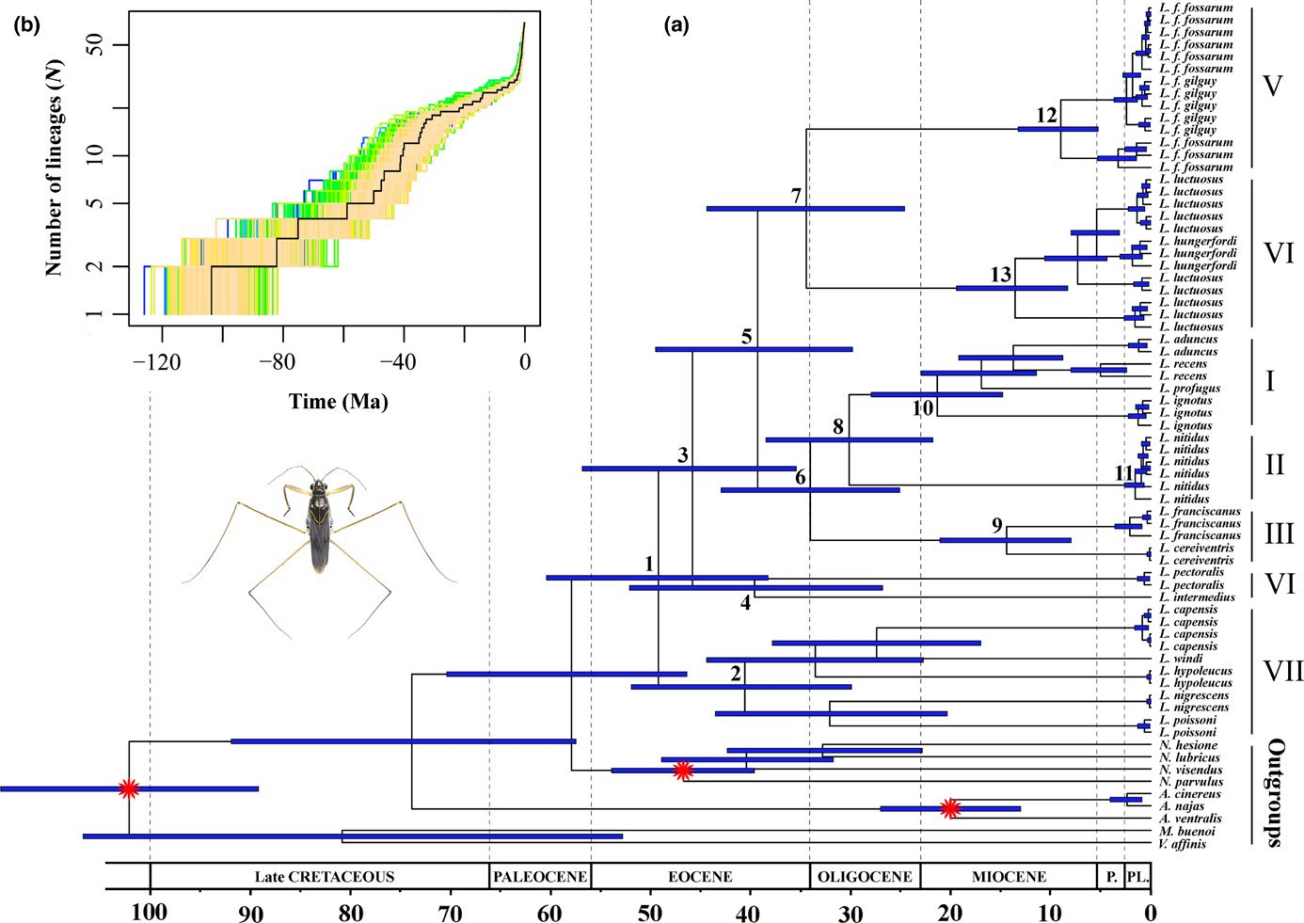
(a) Chronogram of *Limnogonus* was derived from the maximum clade credibility tree estimated from BEAST analyses. The blue bars indicate 95% highest posterior density (HPD) intervals of the age estimates. The red star represents the fossil calibration nodes (see Materials and methods). (b) Semilogarithmic lineage‐through‐time (LTT) plots show the cumulative number of lineages over time represented in (a). The black line shows the maximum clade credibility tree. Uncertainty in age estimation is illustrated by the LTT plots of 1,000 chronograms randomly sampled from the posterior distribution of the MCMC BEAST analyses. This study uses a numbering system with clades I–VII as in the study by Damgaard et al., 2010

The crown group represented ancestor and the origin of diversification. The crown group of *Limnogonus* originated and diversified in the early Eocene at 49 Ma (HPD: 38–60 Ma; node 1; Figure 1a), with subsequent establishment of seven clades during the Eocene to Oligocene (nodes 1→13; Figure 1a). Two split events between the Old World and New World *Limnogonus* were identified: one between clades I and II around the middle Oligocene (30 Ma, HPD: 22–38 Ma; node 8, Figure 1a) and the other in clade III during the middle Miocene (14 Ma, HPD: 8–21 Ma; node 9, Figure 1a).

### 3.2 Diversification analyses

**Figure 2:**
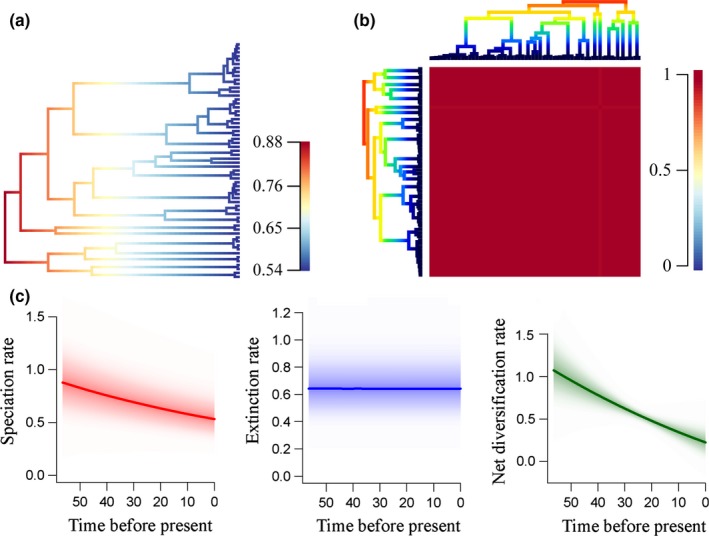
Diversification pattern in *Limnogonus*. (a) The mean phylorate plot shows the diversification rates of cladogenesis of *Limnogonus*. Colors along branches denote instantaneous rates. The warmer (red) colors indicate faster rates than cooler (blue) colors. (b) The macro‐evolutionary cohort matrix for speciation in *Limnogonus*. Every cell in the matrix is coded by a color indicating the pairwise probability between two lineages and their common macro‐evolutionary rate regime. The warmer (red) colors indicate a pairwise probability of 1, whereas cooler (blue) colors mark a pairwise probability of shared macro‐evolutionary dynamics of 0. (c) Speciation, extinction, and net diversification rate over time estimations in *Limnogonus*. The colors polygon denotes 5% through 95% Bayesian credible regions on the distribution of rates

The LTT plots indicated that *Limnogonus* exhibited an almost constant rate of lineage accumulation with a slight increase after the Pliocene (Figure 1b). The mean phylorate plot displayed considerable rate heterogeneity across the phylogenetic tree (Figure 2a). The highest diversification rates were indicated at the base of the phylogram (Figure 2a). The monophyletic seven groups respectively diversified with a relatively lower and decreased diversification rates (Figure 2a). The macro‐evolutionary cohort analyses showed that there was only one general macro‐evolutionary dynamic across *Limnogonus* (Figure 2b). The rate‐through‐time plots indicated moderately decreased speciation and net diversification rates over time, whereas the extinction rates remained almost constant (Figure 2c).

### 3.3 Ancestral area reconstruction

**Figure 3:**
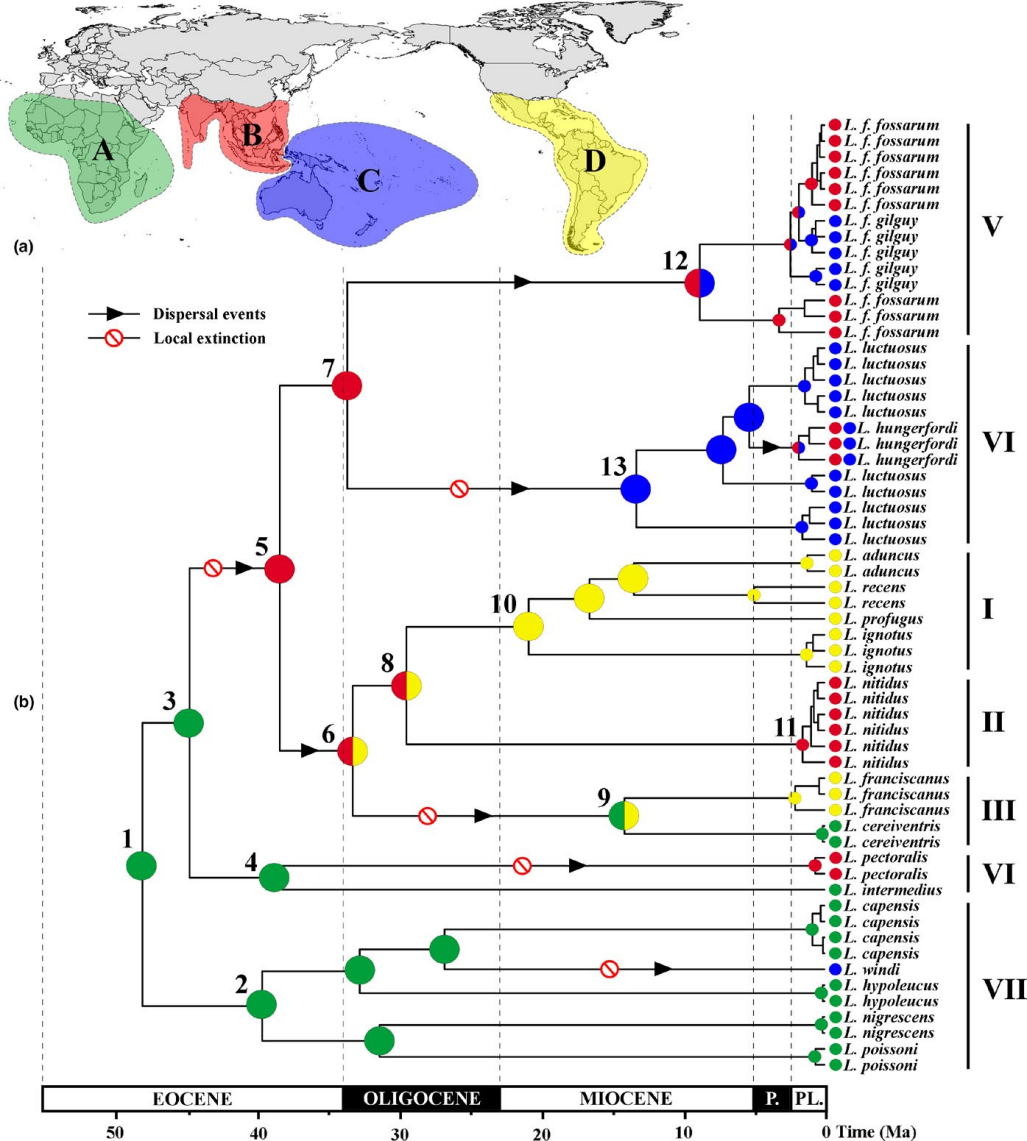
Global biogeographical patterns for *Limnogonus*. (a) Map showing four biogeographical regions in different colors, as defined in this study. Biogeographical region abbreviations: A, Africa; B, India, Indo‐China Peninsula, and Malesia; C, New Guinea, Pacific islands, and Australia; D, Central America, Caribbean, and South America. (b) Schematic chronograms obtained from BEAST show ancestral area reconstruction (AAR) based on the “boreotropical migration” model in the dispersal–extinction–cladogenesis (DEC) model. Colored circles preceding the species names indicate the distribution of extant species. This study uses a numbering system with clades I–VII as in the study by Damgaard et al., 2010

The likelihood ratios, χ^2^, and AIC tests revealed the “boreotropical migration” model could be the optimal biogeographical model for the evolution of *Limnogonus* (*p* < .001; Table S3). The crown group of *Limnogonus* originated and diversified in Africa (node 1; Figure 3b), subsequently expanding into other regions *via* dispersal. A minimum of two dispersals from Africa to the Oriental Region was indicated in the middle to late Eocene (nodes 3 and 4; Figure 3b). The colonization of the New World only originated from the Oriental Region in the late Eocene (node 5; Figure 3b). The crown group of clades I, II, and III diversified in the Oriental and Neotropical Regions with subsequent range expansions into Africa *via* dispersal (node 6; Figure 3b), followed by splits into the Old and New World lineages in the middle Oligocene and middle Miocene, respectively (nodes 8 and 9; Figure 3b). A minimum of two dispersals from the Oriental to Australian Region occurred during the Eocene–Oligocene boundary (node 7; Figure 3b) and formed the clades V and VI, respectively (nodes 12 and 13; Figure 3b).

## 4 Discussion

**Figure 4:**
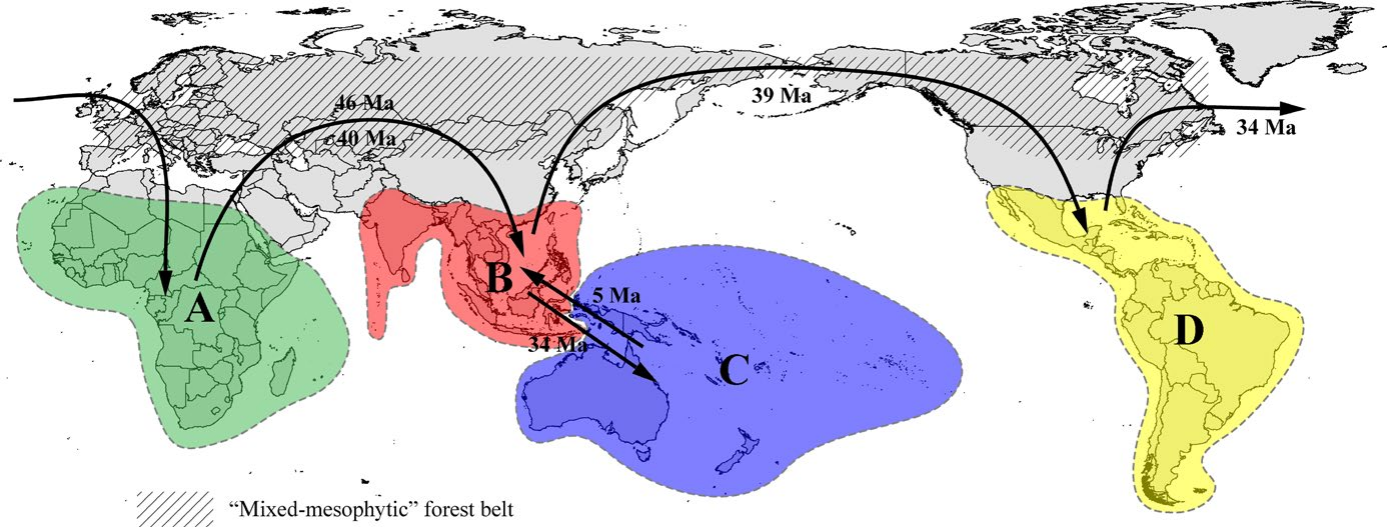
Proposed migration routes suggested by DEC analyses of *Limnogonus* species. The dispersal time estimating from BEAST analyses was also marked. The black arrows represent the dispersal direction

The diversification of the *Limnogonus* crown group was estimated to have occurred in the early Eocene around 49 Ma with a confidence interval of 38–60 Ma. Our combination of divergence time estimates and ancestral area reconstruction supported a probable Africa origin for the *Limnogonus* crown group (node 1; Figure 3b), with subsequent dispersals and diversification into the Oriental, Australian, and Neotropical regions during the Eocene to Miocene. Currently, the global distribution of *Limnogonus* shows a distinct pantropical pattern (Figure 4). However, the estimated age of the diversification of even the oldest confidence interval in the crown group of *Limnogonus* (around 60 Ma) postdates the main breakup sequence of Gondwana (>80 Ma; McLoughlin, 2001; Sanmartín & Ronquist, 2004). Furthermore, in our phylogenetic analyses (Figure 3b), species from the three Gondwanan continents exhibited a much closer relationship with the Oriental Region from Laurasia than each other. In this regard, the hypothesis states that vicariance followed the breakup of Gondwana could not be supported.

Our comparisons of biogeographical models from DEC analyses strongly favoured the “boreotropical migration” model over those of the “LDD” model (*p* < .001; Table S3). In the “boreotropical migration” scenario, migration routes of *Limnogonus* from the Oriental to Neotropical Region *via* the BLB and from the Neotropical to African Region *via* the NALB in the late Eocene could be inferred from our data (Figure 4). The two intercontinental land bridges, BLB and NALB, were critical to the intercontinental exchange of flora or fauna at high latitudes in the Northern Hemisphere, as indicated by a wealth of fossil deposits of shared plants and animals in Eurasia and North America (Viruel et al., 2016). The BLB existed for most of the Tertiary until about 5.5 Ma, although local climatic cooling probably cut off this migration route in advance (Mao et al., 2010). The NALB was present during the early Tertiary but gradually disintegrated at around 50 Ma. After that, plant and animal migrations became difficult, although island chains might have permitted migration for a certain period after the direct land connection disappeared (Mao et al., 2010). Although no fossils were known for *Limnogonus* species in the Northern Hemisphere, only one well‐preserved fossil taxa, *Succineogerris larssoni* Andersen, 2000; was found from Eocene–Oligocene (38–54 Ma) Baltic amber and can be regarded as a close relative of *Neogerris* and *Limnogonus* (Andersen, 2000; Damgaard et al., 2014; Zettel & Heiss, 2011). The rare discoveries of *Limnogonus* fossils from amber might be due to the low fossilization rates of semi‐aquatic insects because their living habitats were semi‐aquatic environments and amber originated from the resin of extinct trees (Zettel & Heiss, 2011). The fossil taxa *S. larssoni* was assumed to inhabit similar lentic habitats of *Neogerris* and *Limnogonus* because of their wing plesiomorphism. In addition, the dispersal time of *Limnogonus* across the Northern Hemisphere from our age estimation was between 34 and 46 Ma (Figure 4), which probably corresponded with the age of *S. larssoni* from Baltic amber (38–54 Ma). Therefore, it was very likely that *Limnogonus* also once inhabited the relatively high latitudes of the Northern Hemisphere during that time.

It was noteworthy that ancestral area reconstruction indicated that the colonization of the New World directly originated from the Oriental Region (nodes 5; Figure 3b) rather than from much closer Australian Region (Figure 4), which also suggested that the diversification of *Limnogonus* probably dispersed through the BLB and migrated into the Neotropical Region rather than by means of direct transoceanic LDD. The dispersal time from the Oriental to Neotropical Region was estimated around 39 Ma (Figure 4), and following that, only speciation events mediated by vicariance were detected around 30 Ma in our model (nodes 8; Figure 3b). This was probably because climatic cooling in the Northern Hemisphere after 34 Ma fragmented the “mixed‐mesophytic” forest belt and decreased the number of suitable water habitats and accordingly cut off the migration routes across the BLB in advance (Wei et al., 2015). The time coincidence was potentially supported by the close relationship between plants and water resource, namely hydrological effects of the forest ecosystem (Brauman, Daily, Duarte, & Mooney, 2007). The forest ecosystem could transfer surface runoff to underground runoff, slowing down the flow velocity. Therefore, in the rainy season, abundant storage water could accumulate and reduce flood flow, while the dry season could supplement the river flow and reduce or prevent drought (Brauman et al., 2007). In addition, ancestral area reconstruction also showed one dispersal event occurred probably *via* the NALB from the Nearctic to the African Region forming clade III (node 9; Figure 3b). Molecular studies using divergence time estimates have provided evidence for *Limnogonus* migration along this route around 34 Ma (Figure 4), which is approximately the limit allowing for dispersals by assistance of the “mixed‐mesophytic” forest belt in the Northern Hemisphere. After that time, a boreotropical extinction occurred in large parts of North America, Europe, and other northern regions as the tropical climate disappeared (Zachos, Pagani, Sloan, Thomas, & Billups, 2001). However, the NALB was already broken and no direct migration routes existed after approximately 50 Ma (Viruel et al., 2016). Thus, plant and animal migrations became considerably more difficult after this time although we could not absolutely rule out dispersal *via* the NALB island chain hypothesis. Alternatively, the direct transoceanic “LDD” model between Africa and the Neotropical Region as a competing scenario at node 6 was also possible and could not be fully ruled out by our findings.

Here, we propose that a most likely scenario led to the diversification of *Limnogonus* in accordance with the “boreotropical migration” model hypothesis (Figure 4). During the middle Eocene to early Oligocene, the wide and prosperous forest belt was continuously distributed throughout the Northern Hemisphere, which was associated with an abundant water habitat environment. The two successful colonizations firstly spread from Africa to the Oriental Region *via* Central Asia around 40 Ma and 46 Ma, respectively (nodes 4 and 3; Figure 3b), followed by one dispersal event from the Oriental to Neotropical Region *via* the BLB around 39 Ma (node 5; Figure 3b), with subsequent range expansions into Africa *via* NALB or direct transoceanic LDD around 34 Ma (node 6; Figure 3b). After the early Oligocene (around 34 Ma), the broad forest belt became fragmented and southward migration from the Northern Hemisphere began due to climatic cooling. This occurrence was simultaneously associated with the disappearance of suitable water habitat in the Northern Hemisphere, resulting in the vicariant occurrence between the Old and New World *Limnogonus*. This could be evidenced by the two speciation events mediated by vicariance between the Old World and New World detected in our model (nodes 8 and 9; Figure 3b). The Oriental species *L. nitidus* diverged from Neotropical species (clade I) around 30 Ma and African species *L. cereiventris* diverged from Neotropical species *L. franciscanus* around 14 Ma. Based on the above analyses, this likely explains why the “boreotropical migration” model might provide a better explanation for the disjunct distribution pattern in pantropical *Limnogonus* than the “LDD” model.

Although similar biogeographical patterns had been found in several other plant and animal groups, such as *Dioscorea* (Viruel et al., 2016), *Ceratolejeunea* (Scheben et al., 2016), Papilionidae (Condamine, Sperling, & Kergoat, 2013), and Choreutidae (Rota et al., 2016), recent studies still emphasize that the “LDD” model should be more critically evaluated when analyzing the global distribution patterns of organisms. The “boreotropical migration” model had been much less discussed in pantropical organisms despite the potential value for explaining their distributions. Perhaps this was because most plants and animals were generally considered to have such direct transoceanic LDD abilities with the use of thermal uplifts and wind (Chapman & Drake, 2010), or ancestral area reconstruction analyses explicitly indicated the direct migration routes between the Australian and Neotropical/African Region (Toussaint et al., 2016), or estimated dispersal ages between the Old and New World were not in accordance with the ages of land bridge connections (BLB or NALB) or did not corresponded with the age of existence of the broad “mixed‐mesophytic” forest belt in the Northern Hemisphere (Scheben et al., 2016). Our study supported the “boreotropical migration” hypothesis that the genus *Limnogonus* might have a boreotropical history based on molecular and paleontological evidence and therefore calls for increased attention to be paid to this scenario in biogeographical studies of pantropical insects.

Compared to the favorite “boreotropical migration” model for the disjunction among African, Oriental, and Neotropical regions, our combination of divergence time estimates and ancestral area reconstruction supported a probable “LDD” model between the Oriental and Australia regions. Two dispersals events from the Oriental to Australian regions occurred around 34 Ma and formed the species *L. fossarum*,* L. luctuosus*, and *L. hungerfordi* (nodes 12 and 13; Figure 3b). During that time, the Australian plate was much farther from the Oriental Region than it is currently. Interestingly, these three species all belong to widespread species, of which, two taxa, *L. fossarum* and *L. hungerfordi*, are currently distributed from Oriental to Australian regions. These widespread species were assumed to be characterized by relatively strong dispersal abilities. Studies had shown that these three *Limnogonus* species were truly wing polymorphic, that is, having both the macropterous morph and two or more morphs with reduced wings (Andersen, 1975). Wing polymorphism leading to intraspecific variation of thoracic structures and the high frequency of the macropterous morph in these three species were probably an adaptation to escape drought and to allow colonization of new and temporary water bodies during the rainy season (Andersen & Weir, 2004), which allowed them to obtain wide geographic distributions. Within the Australian Region, many rare *Limnogonus* species were endemic in the isolated Pacific islands; each of the larger islands was comprised of one or more recent immigrants (e.g., *L. fossarum*,* L*. *luctuosus*, and *L. hungerfordi*) and one endemic species (Damgaard et al., 2010, 2014). These three species inhabiting the islands only differed from the continental population in minute characteristics which indicated their retained wide habitat tolerance (Andersen, 1975). Based on the good dispersal abilities of these three species, the “LDD” model coupled with island hopping could be a reasonable scenario for the diversification of *Limnogonus* between the Oriental and Australian regions. Further studies should comprise more *Limnogonus* taxa from these two regions, especially endemics from the Pacific Island to exactly test this scenario.

## 5 Conclusions

There were few clear examples to rigorously test the hypothesis that the diversification of pantropical insects corresponded with the “boreotropical migration” model. The pond skater genus *Limnogonus* presented a strong case in support of the hypothesis in semi‐aquatic organisms. In this scenario, *Limnogonus* originated and diversified in Africa in the early Eocene, subsequently expanding into the Oriental Region *via* dispersal. The colonization of the New World only originated from the Oriental Region likely *via* the BLB before local cooling during the late Eocene. The modern transoceanic disjunction of *Limnogonus* could be better explained by the disruption of the “mixed‐mesophytic” forest belt although the direct transoceanic LDD between the Neotropics and Africa could not be fully ruled out. The “LDD” model coupled with island hopping could be a reasonable scenario for the diversification between the Oriental and Australian regions during the Oligocene. These findings contributed to our understanding of the distribution and diversification processes of pantropical insects in biogeographical studies.

## Conflict of interest

None declared.
