# Supplementary material for: Out of Africa: Biogeography and diversification of the pantropical pond skater genus Limnogonus Stål, 1868 (Hemiptera: Gerridae)
Source: Ecol Evol. 2017 Jan 1;7(3):793–802. doi: 10.1002/ece3.2688 (PMC5288246; doi:10.1002/ece3.2688)
Supplement: Supplementary file 1 [file ECE3-7-793-s001.doc]

**SUPPORTING INFORMATION**

**Out of Africa: biogeography and diversification of the pantropical pond skater genus *Limnogonus* Stål, 1868 (Hemiptera: Gerridae)**

ZHEN YE, YAHUI ZHEN, YANYAN ZHOU and WENJUN BU

**Table S1** Taxa, voucher information and GenBank accession numbers of specimens used in this study. The defined species follows classification of Andersen (1975, 1995); * means sequences are newly generated in the present study; “–” means missing data. ^1^ including tRNA-Leu gene.

| **Species** | **Location** | **Deposited place** | **GenBank accession numbers** | | | | |
| --- | --- | --- | --- | --- | --- | --- | --- |
|  |  |  | ***COI-COII***^1^ | | ***16S*** | ***28S*** | |
| **Ingroup Taxa** | | | | | | | |
| ***Limnogonus* Stål** | | | | | | | |
| *Limnogonus capensis* | Tanzania, Mt. Rongwe | GenBank | GU645151 | GU645045 | | | GU645100 |
| *Limnogonus capensis* | Tanzania, Mt. Rongwe | GenBank | GU645152 | GU645046 | | | GU645101 |
| *Limnogonus capensis* | South Africa, Buffelspoort Dam | GenBank | GU645153 | GU645047 | | | GU645102 |
| *Limnogonus capensis* | South Africa, Mpummalanga | GenBank | GU645154 | GU645048 | | | GU645103 |
| *Limnogonus hypoleucus* | Tanzania, Minziro Forest Reserve | GenBank | GU645157 | GU645051 | | | – |
| *Limnogonus hypoleucus* | Cameroun, Mbalmayo | GenBank | GU645158 | GU645052 | | | GU645106 |
| *Limnogonus intermedius* | Uganda, Kibale For. Res., Nyabikere | GenBank | GU645159 | GU645053 | | | GU645107 |
| *Limnogonus nigrescens* | Botswana, Okawango Delta | GenBank | GU645160 | GU645054 | | | GU645108 |
| *Limnogonus nigrescens* | Botswana, Okawango Delta | GenBank | GU645161 | GU645055 | | | GU645109 |
| *Limnogonus pectoralis* | Thailand, Pattani Bay | GenBank | GU645178 | GU645072 | | | GU645125 |
| *Limnogonus pectoralis* | Thailand, Pattani Bay | GenBank | GU645179 | GU645073 | | | GU645126 |
| *Limnogonus poissoni* | Tanzania, Mt. Rongwe | GenBank | GU645156 | GU645050 | | | GU645105 |
| *Limnogonus poissoni* | Uganda, Kibale For. Res., Nyabikere | GenBank | GU645155 | GU645049 | | | GU645104 |
| *Limnogonus cereiventris* | Rodrigues, Saint Gabriel | GenBank | GU645170 | GU645064 | | | – |
| *Limnogonus cereiventris* | Rodrigues, Saint Gabriel | GenBank | GU645171 | GU645065 | | | GU645118 |
| *Limnogonus fossarum* | India, Andaman Is. | GenBank | GU645189 | GU645083 | | | GU645136 |
| *Limnogonus f. fossarum* | Laos, Long Nai Kao | GenBank | GU645180 | GU645074 | | | GU645127 |
| *Limnogonus f. fossarum* | Laos, Naten | GenBank | GU645181 | GU645075 | | | GU645128 |
| *Limnogonus f. fossarum* | Maldives, Foammulah Atoll | GenBank | GU645182 | GU645076 | | | GU645129 |
| *Limnogonus f. fossarum* | Thailand, Bangkok | GenBank | GU645183 | GU645077 | | | GU645130 |
| *Limnogonus f. fossarum* | Vietnam, Thvan Hoa | Nankai university, China | KY172130* | KY172123* | | | KY172137* |
| *Limnogonus f. fossarum* | China, Yunnan, Xishuangbanna | Nankai university, China | KY172133* | KY172126* | | | KY172140* |
| *Limnogonus f. fossarum* | China, Hainan, Wuzhi Mt. | Nankai university, China | KY172131* | KY172124* | | | KY172138* |
| *Limnogonus f. fossarum* | China, Taiwan, Pingdong | Nankai university, China | KY172132* | KY172125* | | | KY172139* |
| *Limnogonus f. gilguy* | New Caledonia, Koumac | GenBank | GU645185 | GU645079 | | | GU645132 |
| *Limnogonus f. gilguy* | New Caledonia, Koumac | GenBank | GU645184 | GU645078 | | | GU645131 |
| *Limnogonus f. gilguy* | Australia, WA, Callythara Springs | GenBank | GU645186 | GU645080 | | | GU645133 |
| *Limnogonus f. gilguy* | Australia, QLD, Lakefield N.P. | GenBank | GU645187 | GU645081 | | | GU645134 |
| *Limnogonus f. gilguy* | Australia, NT, Nhulunbuy | GenBank | GU645188 | GU645082 | | | GU645135 |
| *Limnogonus windi* | Australia, WA, Prince Regent River | GenBank | GU645203 | GU645097 | | | GU645148 |
| *Limnogonus franciscanus* | Aruba, Boca Prins | GenBank | GU645175 | GU645069 | | | GU645122 |
| *Limnogonus franciscanus* | Aruba, Masiduri, Arikok N.P. | GenBank | GU645176 | GU645070 | | | GU645123 |
| *Limnogonus franciscanus* | Mexico, Chiapas, Palenque | GenBank | GU645177 | GU645071 | | | GU645124 |
| *Limnogonus recens* | Ecuador, Prov. Esmeraldas Montalvo | GenBank | GU645163 | GU645057 | | | GU645111 |
| *Limnogonus recens* | Honduras, Cortes, Yojoa | GenBank | GU645169 | GU645063 | | | GU645117 |
| *Limnogonus aduncus* | Argentina, Jujuy, Aguas Calientes | GenBank | GU645164 | GU645058 | | | GU645112 |
| *Limnogonus aduncus* | Trinidad, Simla, garden pond 400 m | GenBank | GU645162 | GU645056 | | | GU645110 |
| *Limnogonus ignotus* | Argentina, Chaco, R.V.S. El Cachapé | GenBank | GU645165 | GU645059 | | | GU645113 |
| *Limnogonus ignotus* | Argentina, Chaco, R.V.S. El Cachapé | GenBank | GU645167 | GU645061 | | | GU645115 |
| *Limnogonus ignotus* | Argentina, Corrientes, P. N. Mburucuyá | GenBank | GU645166 | GU645060 | | | GU645114 |
| *Limnogonus profugus* | Argentina, Misiones | GenBank | GU645168 | GU645062 | | | GU645116 |
| *Limnogonus hungerfordi* | Malaysia, Sarawak, Lipad River | GenBank | GU645192 | GU645086 | | | GU645139 |
| *Limnogonus hungerfordi* | Australia, QLD, Cape Tribulation Road | GenBank | GU645194 | GU645088 | | | GU645141 |
| *Limnogonus hungerfordi* | Australia, QLD, 11 km. N Gladstone | GenBank | GU645193 | GU645087 | | | GU645140 |
| *Limnogonus luctuosus* | New Caledonia, Koumac | GenBank | GU645195 | GU645089 | | | GU645142 |
| *Limnogonus luctuosus* | New Caledonia, Col dÁmou | GenBank | GU645199 | GU645093 | | | GU645144 |
| *Limnogonus luctuosus* | New Caledonia, Mandgelia | GenBank | GU645196 | GU645090 | | | GU645143 |
| *Limnogonus luctuosus* | New Caledonia, Auopinié | GenBank | GU645197 | GU645091 | | | – |
| *Limnogonus luctuosus* | New Caledonia, pond W of Yaté | GenBank | GU645198 | GU645092 | | | – |
| *Limnogonus luctuosus* | Australia, NT, Darwin | GenBank | GU645200 | GU645094 | | | GU645145 |
| *Limnogonus luctuosus* | Australia, QLD, Mt. Koo-Thie Bot. | GenBank | GU645201 | GU645095 | | | GU645146 |
| *Limnogonus luctuosus* | Australia, QLD, Mackay | GenBank | GU645202 | GU645096 | | | GU645147 |
| *Limnogonus luctuosus* | French Polynesia, Moorea | GenBank | GU645190 | GU645084 | | | GU645137 |
| *Limnogonus luctuosus* | French Polynesia, Moorea | GenBank | GU645191 | GU645085 | | | GU645138 |
| *Limnogonus nitidus* | India, Andaman Is. | GenBank | GU645172 | GU645066 | | | GU645119 |
| *Limnogonus nitidus* | Maldives Foammulah Atoll | GenBank | GU645173 | GU645067 | | | GU645120 |
| *Limnogonus nitidus* | Vientiane Province | GenBank | GU645174 | GU645068 | | | GU645121 |
| *Limnogonus nitidus* | Vietnam, Tan Hoa | Nankai university, China | KY172134* | KY172127* | | | KY172141* |
| *Limnogonus nitidus* | China, Hainan, Jianling Mt. | Nankai university, China | KY172135* | KY172128* | | | KY172142* |
| *Limnogonus nitidus* | China, Taiwan, Pingdong | Nankai university, China | KY172136* | KY172129* | | | KY172143* |
| **Outgroups** | | | | | | | |
| *Neogerris hesione* | USA, N. Carolina, Chuns Cave | GenBank | KC880956 | | KC880881 | | KC880912 |
| *Neogerris lubricus* | Guyana, Timitiro Airport | GenBank | KC880961 | | KC880882 | | KC880913 |
| *Neogerris parvulus* | Laos, Champasak Province | GenBank | KC880957 | | KC880883 | | KC880914 |
| *Neogerris visendus* | Venezuela, “TVM0200” | GenBank | KC880939 | | KC880880 | | KC880911 |
| *Aquarius cinereus* | Morocco, Azrou Ifrane Area | GenBank | AF200248 | | AY425234 | | DQ683301 |
| *Aquarius najas* | Denmark, Zealand, Lellinge Å | GenBank | AF200736 | | AY425199 | | DQ683302 |
| *Aquarius ventralis* | Bulgaria, Strandzha Mt. | GenBank | AY425249 | | AY425196 | | DQ683300 |
| *Microvelia buenoi* | Denmark | GenBank | EU871298 | | EU871173 | | EU871233 |
| *Velia affinis* | Cyprus | GenBank | EU871319 | | EU871189 | | EU871250 |

**Table S2** Time slices and relative probabilities of dispersal event among regions in (a) “LDD” model and (b) “boreotropical migration” model used in DEC analyses. Geographic area labels as stated in the map (see Fig. 3A): (A) Africa; (B) India, Indo-China Peninsula, and Malesia; (C) New Guinea, Pacific islands and Australia; (D) Central America, Caribbean and South America.

1. “LDD” model (high dispersal)

| Region | A | B | C | D |
| --- | --- | --- | --- | --- |
| A | – | 1.00 | 1.00 | 1.00 |
| B | 1.00 | – | 1.00 | 1.00 |
| C | 1.00 | 1.00 | – | 1.00 |
| D | 1.00 | 1.00 | 1.00 | – |

(b) “boreotropical migration” model (with time slices)

| Region | A | B | C | D |
| --- | --- | --- | --- | --- |
| 0–5 Million years ago (Ma) | | | | |
| A | – | 0.50 | 0.10 | 0.10 |
| B | 0.50 | – | 1.00 | 0.10 |
| C | 0.10 | 1.00 | – | 0.10 |
| D | 0.10 | 0.10 | 0.10 | – |

| Region | A | B | C | D |
| --- | --- | --- | --- | --- |
| 5–30 Million years ago (Ma) | | | | |
| A | – | 0.50 | 0.10 | 0.10 |
| B | 0.50 | – | 0.50 | 0.10 |
| C | 0.10 | 0.50 | – | 0.10 |
| D | 0.10 | 0.10 | 0.10 | – |

| Region | A | B | C | D |
| --- | --- | --- | --- | --- |
| 30–45 Million years ago (Ma) | | | | |
| A | – | 0.75 | 0.10 | 0.10 |
| B | 0.75 | – | 0.25 | 0.10 |
| C | 0.10 | 0.25 | – | 0.10 |
| D | 0.10 | 0.10 | 0.10 | – |

| Region | A | B | C | D |
| --- | --- | --- | --- | --- |
| > 45 Million years ago (Ma) | | | | |
| A | – | 0.75 | 0.10 | 0.10 |
| B | 0.75 | – | 0.10 | 0.10 |
| C | 0.10 | 0.10 | – | 0.50 |
| D | 0.10 | 0.10 | 0.50 | – |

**Table S3** Likelihood-ratio, χ^2^ test and AIC comparison of two dispersal-extinction- cladogenesis (DEC) models (“LDD” model vs. “boreotropical migration” model).

| Models | –ln *L* | χ^2^ | d.f. | *P* | AIC |
| --- | --- | --- | --- | --- | --- |
| ‘LDD’ model | 73.8279 | 12.3452 | 1 | 0.0004* | 151.6558 |
| “boreotropical migration” model | 67.6553 |  | 1 |  | 139.3106 |

**Figure S1** Phylogram of *Limnogonus* obtained from Bayesian inference (BI) analyses and maximum likelihood (ML) of the combined nuclear and mitochondrial markers including sequences of *COI+COII, 16S* and *28S*. Numbers of each branch are support values of the ML and BI analyses in the order of BS_ML_/PP_BI_. The dash (~) indicates a node with BS_ML_ < 50% and PP_BI_ < 0.60. The present study uses a numbering system with clades I-VII as in the paper of Damgaard *et al*., 2010.

**
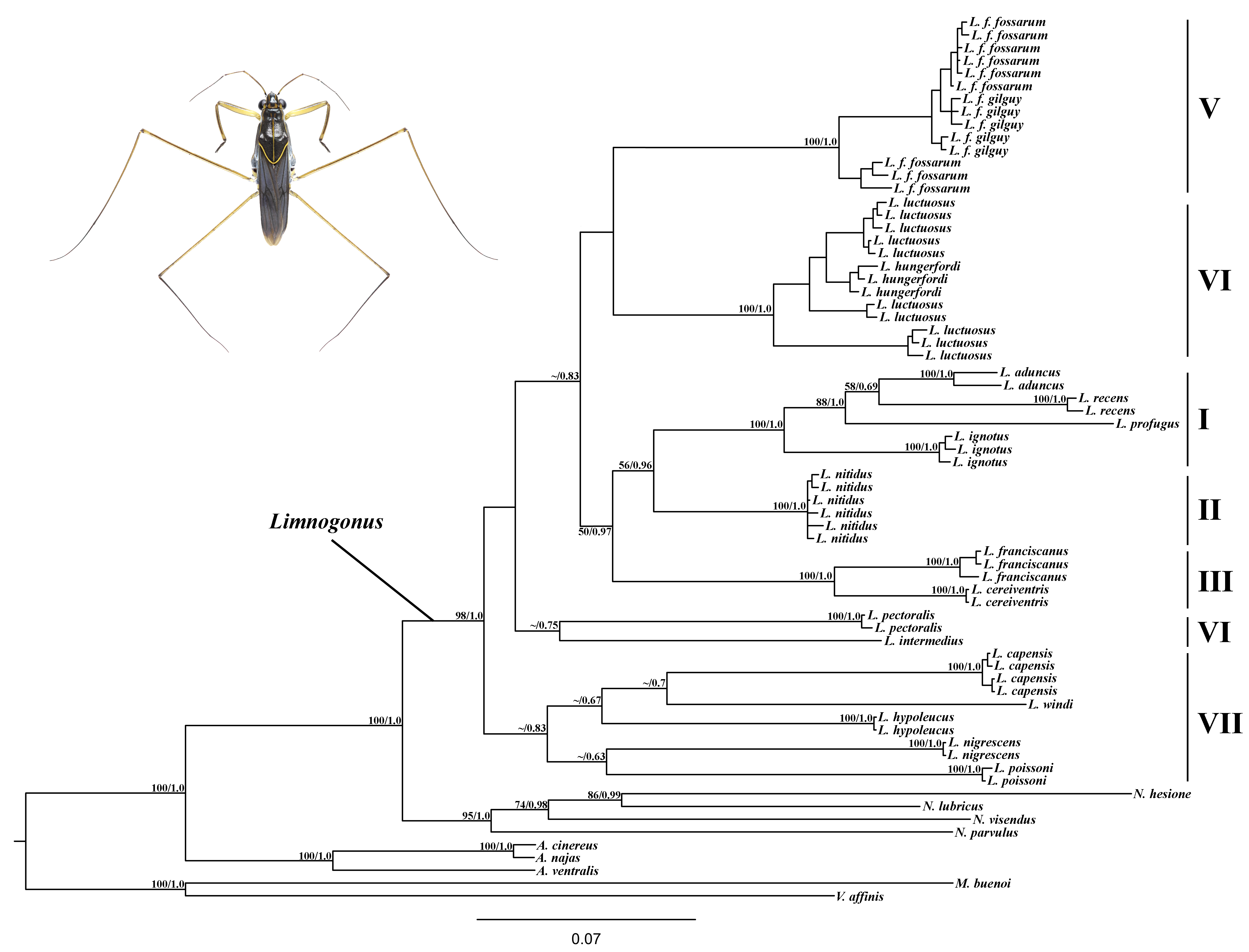
**
